# Ribosomal and Protein Gene Phylogeny Reveals Novel Saprobic Fungal Species From *Juglans regia* and *Urtica dioica*

**DOI:** 10.3389/fmicb.2020.01303

**Published:** 2020-06-30

**Authors:** Dhandevi Pem, Rajesh Jeewon, Faruk Selcuk, Merve Ulukapi, Jayarama Bhat, Mingkwan Doilom, Saisamorn Lumyong, Kevin D. Hyde

**Affiliations:** ^1^Center of Excellence in Fungal Research, Mae Fah Luang University, Chiang Rai, Thailand; ^2^Faculty of Science, Mae Fah Luang University, Chiang Rai, Thailand; ^3^Department of Health Sciences, Faculty of Science, University of Mauritius, Reduit, Mauritius; ^4^Department of Molecular Biology and Genetic, Sciences and Arts Faculty, Kırşehir Ahi Evran University, Kirsehir, Turkey; ^5^Biology Department, Graduate School of Natural and Applied Sciences, Kırşehir Ahi Evran University, Kirsehir, Turkey; ^6^Retired, Goa Velha, India; ^7^Key Laboratory for Plant Diversity and Biogeography of East Asia, Kunming Institute of Botany, Chinese Academy of Sciences, Kunming, China; ^8^World Agroforestry Centre, Kunming, China; ^9^Honghe Center for Mountain Futures, Kunming Institute of Botany, Kunming, China; ^10^Department of Biology, Faculty of Science, Chiang Mai University, Chiang Mai, Thailand; ^11^Research Center of Microbial Diversity and Sustainable Utilization, Chiang Mai University, Chiang Mai, Thailand; ^12^Academy of Science, The Royal Society of Thailand, Bangkok, Thailand

**Keywords:** 3 new species, asexual fungi, Dothideomycetes, morphology, phylogeny

## Abstract

During an ongoing investigation of Ascomycetes from plant substrates, three saprobic species were found from plant substrates. Two new species, *Leptosphaeria regiae* and *Neomicrosphaeropsis juglandis* were isolated from dead branches of *Juglans regia* from Turkey. Another species is introduced herein as *Subplenodomus urticae* sp. nov within the family Leptosphaeriaceae found on *Urtica dioica* in Italy. Multigene phylogenies based on combined LSU, ITS, SSU, and β-tubulin DNA sequence data generated from maximum likelihood and MrBayes analyses indicate that *Leptosphaeria regiae* is related to *L. slovacica* and forms an independent lineage within the genus *Leptosphaeria*. *Subplenodomus urticae* is basal to *S. iridicola* and its establishment as a new species is strongly supported. *Neomicrosphaeropsis juglandis* forms a moderately supported lineage in between *N. italica* and *N. elaeagni* in the Didymellaceae. Full morphological details are provided herein and phylogenetic relationships of the three new species are also discussed.

## Introduction

The bitunicate fungi commonly known as Dothideomycetes is one of the largest group of fungi with a high level of diversity (Zhang et al., 2012; Hyde et al., 2013, 2018, 2019; Tibpromma et al., 2017; Jayasiri et al., 2019). Most of them exist as decomposers, endophytes, epiphytes, fungicolous, lichenized, or lichenicolous fungi in diverse environments (Jeewon et al., 2013, 2017; Phukhamsakda et al., 2016; Doilom et al., 2017; Wanasinghe et al., 2018; Pem et al., 2019a; Phookamsak et al., 2019). They can reproduce either sexually or asexually (Doilom et al., 2014, 2018; Wijayawardene et al., 2014, 2018; Pem et al., 2019c, d). The role of fungi as decomposers is crucial as it helps in recycling of nutrients and releasing enzymes hence maintaining the nutrient compositions of the ecosystem (Hyde and Jeewon, 2003; Hyde et al., 2005; Tang et al., 2005). Decomposers also play vital role in the eco-system such as breakdown of rock to form soils, protection against pathogens and as a food source and alteration of pollutants (Dighton, 2016; Singh et al., 2016). Leptosphaeriaceae is a family in the order Pleosporales (Dothideomycetes, Ascomycota) introduced by Barr (1987) and typified by the genus *Leptosphaeria*. There are 1,800 epithets of Leptosphaeriaceae recorded in Index Fungorum (2020) with the largest number of species (1,669) occurring in the genus *Leptosphaeria* and 130 epithets in Mycobank (2020) but most of them lack molecular data. The family Leptosphaeriaceae is characterized by immersed to superficial ascomata, cylindrical to oblong pedicellate asci and reddish brown or yellowish brown, septate ascospores (Hyde et al., 2013; Ariyawansa et al., 2015; Dayarathne et al., 2015; Wanasinghe et al., 2016). Asexual morph are coelomycetous producing phialidic or annellidic conidiogenous cells (Wijayawardene et al., 2018). Leptosphaeriaceae species differ from other closely related families in the Dothideomycetes by the presence of a scleroplectenchymatous peridium. During our ongoing survey, another species resembling those of the asexual genus *Neomicrosphaeropsis* was observed. The genus *Neomicrosphaeropsis* was introduced by Thambugala et al. (2016) to accommodate four species namely; *N. italica*, *N. novorossica*, *N. rossica*, and *N. tamaricicola.* The type species is *N. italica* and was isolated from dead branches of *Tamarix* (Tamaricaceae) in Italy. Species of *Neomicrosphaeropsis* are pathogens or endophytes (Wijayawardene et al., 2017) and are morphologically characterized by hyaline to light brown, aseptate, obovoid to ellipsoidal conidia (Wijayawardene et al., 2018). The genus *Neomicrosphaeropsis* has been reported to comprise complex species which are morphologically similar but phylogenetically different (Thambugala et al., 2016). *Neomicrosphaeropsis* resembles species of *Microsphaeropsis* which is also accommodated in Didymellaceae as well as species of *Coniothyrium* in having hyaline to light brown, aseptate, obovoid to ellipsoidal, smooth-walled conidia (Verkley et al., 2014; Crous et al., 2019). There are currently 10 epithets in the genus *Neomicrosphaeropsis* (Index Fungorum, 2020). In this study, we introduce a new asexual species in the genus *Neomicrosphaeropsis* isolated from stems of *Juglans regia* (Juglandaceae) in Turkey using multi-gene (LSU, ITS, SSU, and β-tubulin) phylogenetic data. To the best of our knowledge, there have been no fungal species of Leptosphaeriaceae and Didymellaceae associated with *Juglans regia* in Turkey. We also report on a new species of Leptosphaeriaceae specifically in the genus *Subplenodomus* found on *Urtica dioica* from Italy. The aim of this study is to characterize these three fungal isolates in terms of morphology and phylogeny based on multi-gene sequence data.

## Materials and Methods

### Samples Collection, Morphological Examination, and Isolation

**FIGURE 1:**
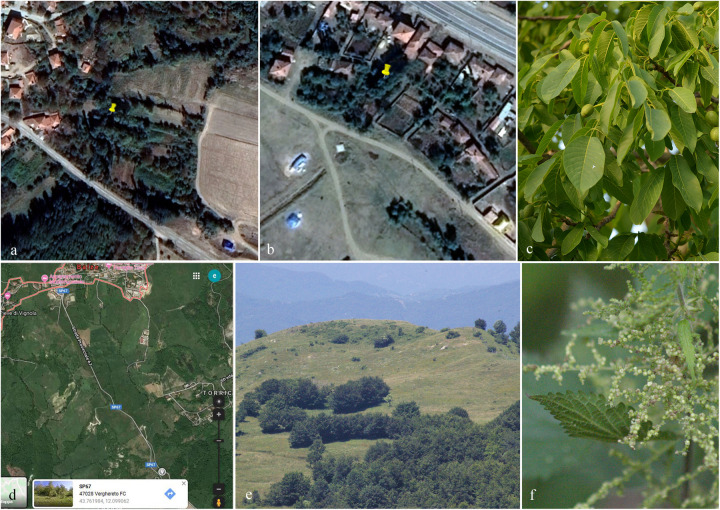
Geographical coordinates and collecting site of specimen (**a,b**: Turkey, **c:** *Juglans regia*; **d,e**: Italy, **f:** *Urtica dioica*).

Specimens were collected from dead stems and branches of *Juglans regia* in the Corum and Kirikkale province of Turkey and on *Urtica dioica* in the province of Forlì-Cesena (FC) Italy (Figure 1). Samples were stored in Zip-lock bags and returned to the laboratories for examination and description of morphological characters. The specimens were observed under a Motic SMZ 168 series dissecting stereo-microscope. Free hand sections of fungal structures were taken and mounted in water for microscopic study. Photomicrography was carried out using a Canon 750D digital camera fitted to the microscope. Measurements were made with the Tarosoft (R) Image Frame Work software. The images were processed with Adobe Photoshop CS5 v. 12.0 software (Adobe Systems, United States) to illustrate fungal characters using a photoplate. Pure cultures were established from single ascospores/conidia on 2% malt extract agar (MEA; 62 g/L Criterion in distilled water) as described in Vijaykrishna et al. (2004) and Pem et al. (2019d). Cultures were incubated at 25°C for up to 5 weeks and cultural characters were observed and measured after a week and again after 4 weeks following Liu et al. (2014). Holotype specimens are deposited in the herbarium located at Mae Fah Luang University (MFLU) and isotype specimens are deposited at the Kunming Institute of Botany, Academia Sinica Herbarium (HKAS), China. Ex-type living cultures are deposited at the Mae Fah Luang Culture Collection (MFLUCC) and duplicates at the Leibniz Institute DSMZ-German Collection of Microorganisms and Cell cultures (DSMZ). Faces of fungi numbers (Jayasiri et al., 2015) and Index Fungorum number^1^ are provided.

### DNA Extraction, Amplification, and Sequencing

Isolates were grown on MEA at 16 ± 2°C for 8 weeks. DNA was extracted from fresh fungal mycelium using the DNA extraction kit (E.Z.N.A Fungal DNA Mini Kit, D3390-02, Omega Bio-Tek) following the manufacturer’s protocol. Polymerase Chain Reaction (PCR) was performed to amplify specific gene regions. Primers LR0R and LR5 were used to amplify part of the nuclear ribosomal large subunit 28S rRNA gene (LSU) (Vilgalys and Hester, 1990; Rehner and Samuels, 1994). The Internal transcribed spacer rDNA region (ITS1, 5.8S rDNA, and ITS2) was determined using the primer ITS5/ITS4 and the 18S small subunit ribosomal RNA (SSU) was amplified using NS1/NS4 (White et al., 1990). For the beta-tubulin (TUB) gene, partial cds region, the primers Bt2a/Bt2b was used (Woudenberg et al., 2009). Primer sequences are available at the WASABI (Web Accessible Sequence Analysis for Biological Inference) database at the AFTOL website (aftol.org). Amplification reactions profiles for LSU, ITS, SSU, and β-tubulin gene followed Tang et al. (2007), Wang et al. (2007), and Luo et al. (2017). The analysis of PCR amplification products (amplimers) were performed by the commercial sequencing provider (BGI, Ltd Shenzhen, PR China). Same primer pairs used for amplification process were used for sequencing. The nucleotide sequence data acquired is deposited in GenBank (Table 1). The final alignment and tree are deposited in the TreeBASE repository site^2^. The recommendations outlined by Jeewon and Hyde (2016) were used to establish the new taxa.

**TABLE 1:** Isolates used in this study. Newly generated sequences are indicated in bold.

Taxon	Strain/Culture No.	Status	GenBank accession numbers			
			LSU	ITS	SSU	BTUB
*Alloleptosphaeria italica*	MFLUCC 14-0934		KT454714	KT454722		
*Allophoma labilis*	CBS 124.93		GU238092	GU237868		GU237620
*Alternariaster bidentis*	CBS 134021	T	KC609341	KC609333		
*Alternariaster centaureae diffusae*	MFLUCC 14-0992	T	KT454715	KT454723	KT454730	
*Alternariaster helianthi*	CBS 327.69	T	KC584369	KC609335	KC584627	
*Alternariaster trigonosporus*	MFLU 15-2237	T	KY674858	KY674857		
*Ascochyta pisi*	CBS 126.54		MH868800	MH857263	EU754038	GU237531
*Ascochyta pisi*	CBS 122751		KP330444	KP330432		KP330388
*Ascochyta pisi*	CBS 122785	T	GU237969	GU237763	EU754039	GU237532
*Boeremia exigua var exigua*	CBS 431.74		JX681074	FJ427001	EU754084	FJ427112
*Boeremia exigua var pseudolilacis*	CBS 101207		GU237941	GU237721		GU237503
*Briansuttonomyces eucalypti*	CBS 114887		KU728520	KU728480		KU728596
*Briansuttonomyces eucalypti*	CBS 114879	T	KU728519	KU728479		KU728595
*Calophoma aquilegiicola*	CBS 108.96		GU238042	GU237736		GU237582
*Calophoma aquilegiicola*	CBS 107.96		GU238041	GU237735		GU237581
*Calophoma clematidina*	CBS 108.79		MH872951	FJ426989		
*Calophoma clematidis-rectae*	CBS 507.63		MH869956	FJ515606		FJ515624
*Calophoma glaucii*	CBS 112.96		GU238077	GU237750		GU237610
*Calophoma vodakii*	CBS 173.53	T	MH868686	MH857149		KT389791
*Cumuliphoma indica*	CBS 654.77		GU238122	FJ427043		FJ427153
*Cumuliphoma indica*	CBS 991.95		GU238121	FJ427044		FJ427154
*Cumuliphoma omnivirens*	CBS 341.86	T	LT623214	MH861962		FJ427152
*Cumuliphoma pneumoniae*	CBS 142454		LN907392	LT592925		T592994
*Didymella aliena*	CBS 379.93		GU238037	GU237851		GU237578
*Didymella anserina*	CBS 360.84		GU237993	GU237839		GU237551
*Didymella chenopodii*	CBS 128.93		GU238055	GU237775		GU237591
*Didymella exigua*	CBS 183.55	T	MH868977	MH857436	GU296147	GU237525
*Didymella glomerata*	DAOM 214575		JN938876	JN942893	JN939031	
*Didymella glomerata*	CBS 464.97		GU238009	FJ427012	EU754086	FJ427123
*Didymella negriana*	CBS 358.71		MH871931	GU237838		GU237635
*Didymella* sp.	CPC 21698		KF777232	KF777180		
*Didymella* sp.	CPC 22587		KJ869191	KJ869134		KJ869246
*Didymellocamarosporium tamaricis*	MFLUCC 15-0067	T	KY496733	KY496753	KY501116	
*Didymellocamarosporium tamaricis*		T	KU848183		KU848182	
*Didysimulans italica*	MFLUCC 15-0059		KY496730	KY496750	KY501118	
*Didysimulans mezzanensis*	MFLUCC 15-0078		KY496733	KY496753	KY501116	
*Dothidotthia aspera*	CPC 12933		EU673276	MK442601	EU673228	
*Dothidotthia symphoricarpi*	CBS 119687		EU673273	MH863064	EU673224	
*Ectophoma multirostrata*	CBS 110.79		GU238110	FJ427030		FJ427140
*Ectophoma multirostrata*	CBS 274.60	T	MH869536	MH857982		FJ427141
*Ectophoma multirostrata*	CBS 368.65		MH870257	MH858615		FJ427143
*Ectophoma pomi*	CBS 267.92	T	GU238128	GU237814		GU237643
*Epicoccum nigrum*	CBS 173.73	T	MH872357	MH860655	GU238206	FJ427107
*Heterophoma novae-verbasicola*	CBS 127.93	T	GU238120	GU237774		GU237639
*Heterophoma sylvatica*	CBS 874.97		GU238147	GU237907		GU237662
*Heterospora chenopodii*	CBS 115.96		EU754188	JF740227	EU754089	
*Heterospora chenopodii*	CBS 448.68	T	EU754188	JF740227	EU754089	
*Heterospora dimorphospora*	CBS 165.78		JF740281	JF740204	JF740098	
*Heterospora dimorphospora*	CBS 345.78		GU238069	JF740203	GU238213	
*Juxtiphoma eupyrena*	CBS 374.91		GU238072	FJ426999		FJ427110
*Juxtiphoma eupyrena*	CBS 527.66		MH870524	FJ427000		FJ427111
*Leptosphaeria cichorium*	MFLUCC 14-1063		KT454712	KT454720	KT454728	
*Leptosphaeria conoidea*	CBS 616.75		MH872726	MH860957	JF740099	KT389804
*Leptosphaeria doliolum*	CBS 505.75	T	GU301827	MH860947	GU296159	JF740144
*Leptosphaeria doliolum*	MFLUCC 15-1875		MH870536	MH858879	GU296159	
*Leptosphaeria ebuli*	MFLUCC 14-0828	T	KP744488	KP744446	KP753954	
*Leptosphaeria errabunda*	CBS 617.75		JF740289	JF740216		JF740150
*Leptosphaeria italica*	MFLU 15-0174		KT783670			
*Leptosphaeria macrocapsa*	CBS 640.93		JF740304	JF740237		JF740156
*Leptosphaeria pedicularis*	CBS 390.80		JF740294	JF740224		JF740155
***Leptosphaeria regiae***	**MFLUCC 18-1137**	**T**	**MN244171**	**MN244201**	**MN244177**	
*Leptosphaeria sclerotioides*	CBS 148.84		JF740270	JF740193		
*Leptosphaeria slovacica*	CBS 125975		JF740316	JF740248		
*Leptosphaeria sydowii*	CBS 385.80		JF740313	JF740244		JF740157
*Leptosphaeria urticae*	MFLU 18-0591	T	MK123332	MK123333	MK123329	
*Leptosphaeria veronicae*	CBS 126583		MH875625	MH864163		JF740161
*Leptosphaerulina arachidicola*	CBS 275.59		MH869401	MH857863		GU237543
*Leptosphaerulina australis*	CBS 317.83		MH873322	MH861604	GU296160	GU237540
*Leptosphaerulina trifolii*	CBS 235.58		MH869300	MH857767		GU237542
*Loratospora aestuarii*	JK 5535B		GU301838		GU296168	
*Macroventuria anomochaeta*	CBS 502.72		MH872250	GU237873		GU237545
*Macroventuria anomochaeta*	CBS 525.71		MH872013	GU237881	GU238208	GU237544
*Macroventuria wentii*	CBS 526.71	T	MH872014	MH860250		GU237546
*Microsphaeropsis olivacea*	CBS 432.71		GU237987	GU237863		GU237548
*Microsphaeropsis olivacea*	CBS 442.83		EU754171	GU237865	EU754072	GU237547
*Microsphaeropsis olivacea*	CBS 233.77		GU237988	GU237803		GU237549
*Microsphaeropsis ononidicola*	MFLUCC 15-0459, ICMP 21575	T	MG967668	MG967670		MG973087
*Microsphaeropsis proteae*	CBS 111303		JN712561	JN712495		
*Microsphaeropsis proteae*	CBS 111320		JN712562	JN712496		JN712649
*Microsphaeropsis proteae*	CBS 111319	T	JN712563	JN712497		JN712650
*Microsphaeropsis spartii-juncei*	MFLU 16-0100	T	MH069668	NR160346	MH069674	MH069687
*Microsphaeropsis spartii-juncei*	MFLU 16-0097		MH069669	MH069663	MH069675	MH069688
*Neoas. europaea*	CBS 820.84	T	KT389729	NR136131		KT389809
*Neoas. exitalis*	CBS 389.86		KT389733	MH861971		KT389813
*Neodid. cannabis*	CBS 121.75	T	GU237972	GU237761		GU237535
*Neodid. polemonii*	CBS 109181	T	GU238133	GU237746		GU237648
*Neodid. xanthina*	CBS 383.68		GU238157	GU237855		GU237669
*Neoleptosphaeria jonesii*	MFLUCC 16-1442	T	KY211870	KY211869	KY211871	
*Neoleptosphaeria rubefaciens*	CBS 223.77	T	JF740312	JF740243		
*Neoleptosphaeria rubefaciens*	CBS 387.80		JF740311	JF740242		
*Neomicrosphaeropsis alhagi-pseudalhagi*	MFLUCC 17-0825	T	MH069670	MH069664	MH069676	MH069689
*Neomicrosphaeropsis cystisi*	MFLUCC 13-0396	T	KX572342	KX572337	KX572347	
*Neomicrosphaeropsis cystisicola*	MFLUCC 18-0355	T	MH069671	MH069665		MH069690
*Neomicrosphaeropsis cystisinus*	MFLUCC 16-0790	T	KX611241	KX611243	KX611242	
*Neomicrosphaeropsis elaeagni*	MFLUCC 17-0740	T	MH069672	MH069666	MH069678	MH069691
*Neomicrosphaeropsis italica*	MFLUCC 15-0485	T	KU729854	KU900318	KU900309	
*Neomicrosphaeropsis italica*	MFLUCC 16-0284		KU900296	KU900321	KU900311	KX453299
*Neomicrosphaeropsis italica*	MFLUCC 15-0484		KU729853	KU900319		KX453298
*Neomicrosphaeropsis italica*	MFLUCC 15-0487		KU729852	KU900320	KU900310	
***Neomicrosphaeropsis juglandis***	**MFLUCC 18-0795**	**T**	**MN244206**	**MN244223**	**MN244183**	**MN871954**
*Neomicrosphaeropsis minima*	MFLUCC 13-0394		KX572341	KX572336	KX572346	
*Neomicrosphaeropsis novorossica*	MFLUCC 14-0578	T	KX198710	KX198709	KX198711	
*Neomicrosphaeropsis rossica*	MFLUCC 14-0586	T	KU729855	KU752192	KU870914	
*Neomicrosphaeropsis tamaricicola*	MFLUCC 14-0443		KU729851	KU900322	KU900312	
*Neomicrosphaeropsis tamaricicola*	MFLUCC 14-0439		KU729858	KU900323	KU900313	
*Neomicrosphaeropsis tamaricicola*	MFLUCC 14-0602	T	KM408754	KM408753	KM408755	MH069692
*Nothophoma anigozanthi*	CBS 381.91	T	GU238039	GU237852		GU237580
*Nothophoma arachidia-hypogaeae*	CBS 125.93		MH874048	MH862388		GU237583
*Nothophoma infossa*	CBS 123395	T	GU238089	MH863295		FJ427135
*Nothophoma infossa*	CBS 123394		GU238088	FJ427024		FJ427134
*Ophiosphaerella herpotricha*	CBS 620.86		DQ678062	KF498728	DQ678010	
*Paraboeremia adianticola*	CBS 187.83		GU238035	GU237796		GU237576
*Paraboeremia putaminum*	CBS 130.69		MH871005	MH859273		GU237652
*Paraboeremia selaginellae*	CBS 122.93	T	GU238142	NR 135980		GU237656
*Paraleptosphaeria dryadis*	CBS 643.86		MH873696	MH862007	KC584632	
*Paraleptosphaeria macrospora*	CBS 114198		MH874520	MH862957		
*Paraleptosphaeria nitschkei*	CBS 306 51	T	MH868393	MH856873		KT389833
*Paraleptosphaeria padi*	MFLU 15-2756	T	KY554198	KY554203	KY554201	
*Paraleptosphaeria rubi*	MFLUCC 14-0211	T	KT454718	KT454726	KT454733	
*Paraphoma radicina*	CBS 111.79		MH872952	MH861183	EU754092	KF252667
*Phaeosphaeria elongata*	CBS 120250		GU456327	MH863080	GU456306	
*Phaeosphaeria oryzae*	CBS 110110	T	MH874442	MH862850	GQ387530	KF252680
*Phaeosphaeriopsis glaucopunctata*	MFLUCC 13-0265		KJ522477	KJ522473	KJ522481	
*Phoma herbarum*	CBS 615.75		KF251715	KF251212	EU754087	KF252703
*Phoma herbarum*	CBS 274.37		KT389754	KT389537		KT389835
*Phoma herbarum*	CBS 502.91		GU238082	GU237874		GU237613
*Phoma neerlandica*	CBS 134.96		KT389753	KT389535		KT389834
*Phomatodes aubrietiae*	CBS 627.97	T	MH874272	GU237895		GU237585
*Phomatodes aubrietiae*	CBS 383.67		GU238044	GU237854		GU237584
*Phomatodes nebulosa*	CBS 127776		MH876211	MH864771		GU237634
*Plenodomus agnitus*	CBS 121. 89		MH875626	KP744459		KY064053
*Plenodomus artemisiae*	KUMCC 18-0151	T	MK387958	MK387920	MK387928	
*Plenodomus chrysanthemi*	CBS 539.63	T	MH869970	MH858349	GU238230	KY064055
*Plenodomus collinsoniae*	CBS 120227		JF740276	JF740200		KY064056
*Plenodomus collinsoniae*	VT2	T		MN653010		
*Plenodomus congestus*	CBS 244.64	T	JF740278	AF439460		KY064058
*Plenodomus deqinensis*	CGMCC 3.18221	T	KY064031	KY064027		KY064052
*Plenodomus enteroleucus*	CBS 142.84	T	JF740287	JF740214		KT266266
*Plenodomus fallaciosus*	CBS 414.62		MH869793	JF740222		
*Plenodomus guttulatus*	MFLUCC 15-1876	T	KT454713	KT454721	KT454729	
*Plenodomus hendersoniae*	CBS 113702		MH874506	MH862939		KT266271
*Plenodomus influorescens*	CBS 143.84		JF740297	JF740228		KT266267
*Plenodomus libanotidis*	CBS 113795		MH874508	MH862943		KY064059
*Plenodomus lijiangensis*	KUMCC 18-0186	T	MK387959	MK387921	MK387929	
*Plenodomus lindquistii*	CBS 381.67		MH870699	MH858999		
*Plenodomus lingam*	CBS 260.94	T	JX681096	MH862462		KY064060
*Plenodomus lupini*	CBS 248.92		JF740303	JF740236		KY064061
*Plenodomus pimpinellae*	CBS 101637		MH874352	JF740240		KY064062
*Plenodomus salviae*	MFLUCC 13-0219	T	KT454717	KT454725	KT454732	
*Plenodomus sinensis*	KUMCC 18-0152		MK387961	MK387923	MK387931	
*Plenodomus sinensis*	KUMCC 18-0153		MK387960	MK387922	MK387930	
*Plenodomus sinensis*	KUN HKAS 102227		MK387962	MK387924	MK387932	
*Plenodomus sinensis*	MFLUCC 17-0757		MF072718	MF072722	MF072720	
*Plenodomus tracheiphilus*	CBS 127250		JF740318	JF740250		
*Plenodomus visci*	CBS 122783	T	EU754195	JF740256	EU754096	KY064063
*Plenodomus wasabiae*	CBS 120119			JF740257		KT266272
*Pseudoascochyta novae-zelandiae*	CBS 141689		LT592893	LT592892		LT592894
*Pseudoleptosphaeria etheridgei*	CBS 125980		MH875320	JF740221		
*Remotididymella anthropophila*	CBS 142462			LT592936		LT593005
*Remotididymella desctructiva*	CBS 378.73	T	MH872414	MH860707		GU237601
*Remotididymella desctructiva*	CBS 133.93		GU238064	GU237779		GU237602
*Remotididymella desctructiva*	CBS 162.78		GU238062	GU237788		GU237600
*Setomelanomma holmii*	CBS 110217		GU301871	KT389542	GU296196	
*Similiphoma crystallifera*	CBS 193.82	T	GU238060	GU237797		GU237598
*Sphaerellopsis filum*	CBS 234.51		KP170723	KP170655		KP170704
*Sphaerellopsis hakeae*	CPC 29566	T	KY173555	KY173466		
*Sphaerellopsis isthmospora*	HKAS 102225A	T	MK387963	MK387925	MK387933	
*Sphaerellopsis isthmospora*	HKAS 102225B	T	MK387964	MK387926	MK387934	
*Sphaerellopsis macroconidiale*	CBS 233.51		KP170726	KP170658		KP170707
*Sphaerellopsis macroconidiale*	CBS 658.78		MH868352	KP170659		KP170708
*Sphaerellopsis paraphysata*	CPC 21841	T	KP170729	KP170662		KP170710
*Stagono. cucurbitacearum*	CBS 133.96		GU238181	GU237780	GU238234	GU237686
*Stagono. hortensis*	CBS 104.42		GU238198	MH856097		GU237703
*Subplenodomus apiicola*	CBS 285.72		GU238040	MH860477	GU238211	
*Subplenodomus drobnjacensis*	CBS 269.92		JF740285	JF740211	JF740100	
*Subplenodomus galicola*	MFLU 15-1368	T	KY554199	MF467894		
*Subplenodomus iridicola*	CBS 143395	T	MH107965	MH107919		
***Subplenodomus urticae***	**MFLUCC 17-2311**	**T**	**MN597995**	**MN597998**	**MN597997**	
*Subplenodomus valerianae*	CBS 630.68		MH870914	JF740251	GU238229	
*Subplenodomus violicola*	CBS 306.68		MH870849	MH859138	GU238231	KT389849
*Vacuiphoma bulgarica*	CBS 357.84	T	GU238050	GU237837		GU237589
*Vacuiphoma oculihominis*	URTHSC D116-308	T	LN907451	LT592954		LT593023
*Xenodidymella applanata*	CBS 195.36	T	KT389764	MH855770		KT389852

*Newly generated sequences are indicated in bold. CBS, Centraalbureau voor Schimmelcultures, Utrecht, The Netherlands; CPC, Collection of Pedro Crous housed at CBS; DAOM, Plant Research Institute, Department of Agriculture (Mycology), Ottawa, Canada; HKAS, the Herbarium of Cryptogams Kunming Institute of Botany Academia Sinica, Kunming, China; JK, J. Kohlmeyer; KUMCC, Kunming Institute of Botany Culture collection, Yunnan, China; MFLUCC, Mae Fah Luang University Culture Collection, ChiangRai, Thailand. Type strains are indicated by “T.”*

### Phylogenetic Analysis

SeqMan v. 7.0.0 (DNASTAR, Madison, WI, United States) was used to assemble consensus sequences. Sequences of closely related strains were recovered from BLAST searches of GenBank^3^ together with sequences of representative species used by Chen et al. (2015), Thambugala et al. (2016), and Phookamsak et al. (2019) and these are listed in Table 1. Sequences were aligned with online MAFFT v. 7 (Kuraku and Katoh, 2013; Katoh et al., 2019)^4^. The alignments were checked visually and improved manually where necessary using BioEdit v. 7.0.5.2 (Hall, 1999). Ambiguous regions were excluded from the analyses and gaps were treated as missing data. All novel sequences were deposited in GenBank and the final alignment and tree deposited in TreeBASE^2^. Phylogenetic analyses were based on maximum likelihood (ML) and Bayesian inference (BI) methods. Maximum likelihood analyses (ML), for single and combined gene alignments included 1,000 bootstrap replicates and was performed using RAxML-HPC2 run on XSEDE (8.2.8) (Stamatakis, 2014) in the CIPRES Science Gateway platform (Miller et al., 2011) using GTR+I+G model of evolution. The final tree was selected among suboptimal trees from each run by comparing likelihood scores with the GTRGAMMA nucleotide substitution model. The best fitting substitution model for each single gene partition and the concatenated data set was determined in MrModeltest 2.3 (Nylander, 2004) for Bayesian inference posterior probabilities (PP). GTR+I+G model was used for each partition or each gene separately, and incorporated into the analysis. The Bayesian inference posterior probabilities (PP) distribution (Zhaxybayeva and Gogarten, 2002) was estimated by Markov Chain Monte Carlo sampling (MCMC) in MrBayes v. 3.2.2 (Ronquist et al., 2012). The MCMC analyses, with six chains were run, started from random tree topology and lasted 1,000,000 generations and sampled every 100 generations (Nylander et al., 2008). The Tracer v. 1.5.0 software program was used to calculate the distribution of log-likelihood scores in order to determine the stationary phase for each search, to check whether extra runs were required to achieve convergence, the stable likelihood plateaus and burn–in value (Drummond et al., 2012). The first 2,000 generations were excluded as burn-in and 10,000 trees were obtained. Maximum likelihood bootstrap values equal or greater than 50% and Bayesian inference posterior probabilities (PP) equal or greater than 0.90 are given in black below or above each node (Figure 1). The phylograms were viewed in FigTree v1.4^5^ and edited using Microsoft PowerPoint 2016.

## Results

### Taxonomy

**FIGURE 2:**
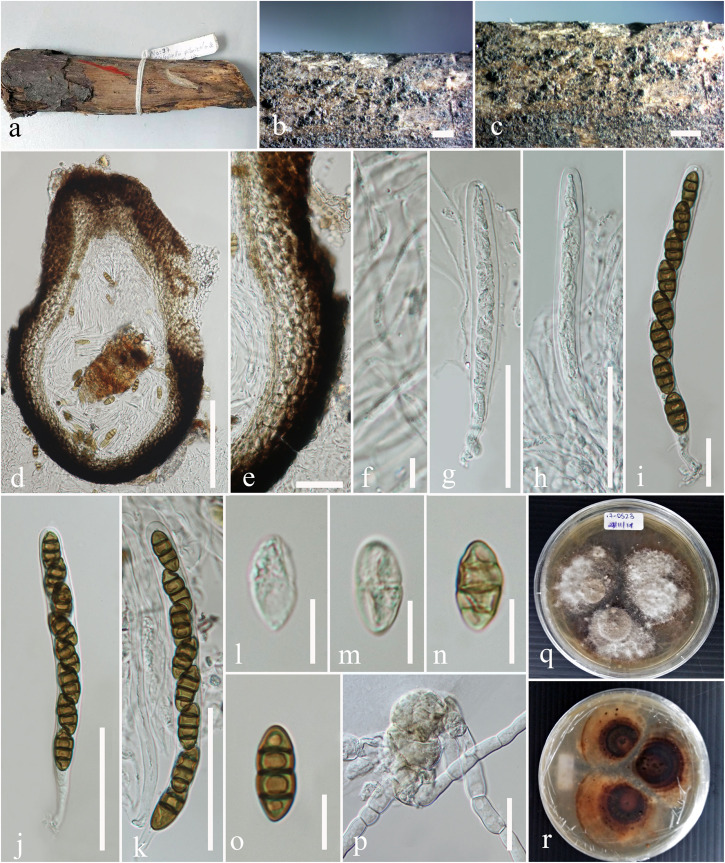
*Leptosphaeria regiae* (MFLU 17-0523, holotype). **(a)** Specimen. **(b,c)** Appearance of ascomata on host surface. **(d)** Vertical section through the ascoma. **(e)** Peridium. **(f)** Hamathecium**. (g**–**k)** Asci. **(l**–**o)** Ascospores. **(p)** Germinating ascospore. **(q,r)** Culture characteristics on MEA (**q:** above view; **r:** reverse view). Scale bars: **(b–d)** = 200 μm, **(e,g,h,k)** = 50 μm, **(f)** = 5 μm, **(i,j)** = 20 μm, **(l–o)** = 10 μm.

***Leptosphaeria regiae*** D. Pem, Selcuk, Jeewon & K.D. Hyde, **sp. nov.** Figure 2

[urn:lsid:indexfungorum.org:names: 557056]
Facesoffungi Number: FoF 06229
Etymology – Name reflects the host from which the fungus was isolated.
Holotype – MFLU 17-0523

*Saprobic* on dead stem of *Juglans regia*. **Sexual morph:** *Ascomata* 315–377 μm high, 364–410 μm diam., solitary or gregarious, superficial or semi-immersed on host tissue, visible as black spots on host surface, brown to dark brown. *Ostiole* apex dark brown to black, ostiolar canal filled with periphyses, papilla not conspicuous. *Peridium* 41–50 μm wide, comprising two cell types, outer layer composed of large, heavily pigmented, thick-walled cells of *textura angularis*, inner layer composed of scleroplectenchymatous cells of *textura angularis*. *Hamathecium* comprising numerous, 1.4–2.6 μm diam., slime coated, branched, cellular pseudoparaphyses. *Asci* 99–130 × 9–10 μm ($\bar{x}$ = 104.8 × 9.9 μm, *n* = 30), 8-spored, bitunicate, numerous, cylindrical to cylindric-clavate, short pedicellate, apically rounded, with indistinct ocular chamber. *Ascospores* 15–18 × 6–7 μm ($\bar{x}$ = 17.1 × 7.1 μm, *n* = 30), uni to bi-seriate, hyaline brown when immature, becoming yellowish brown to brown at maturity, ellipsoid to broadly fusiform, with rounded to acute ends, slightly clavate, narrow toward the base, 3-septate, constricted at septum, widest above the central septum, smooth-walled. **Asexual morph:** Undetermined.

Culture characteristics – Colonies on MEA, 17–20 mm diam. after 7 days at 16°C, margin irregular, aerial mycelia thinly hairy, sparse, white and flat; reverse dark brown, white at the margin.

Material examined – TURKEY, The Middle Kizilirmak river basin, Kirşehir province, Kaman district, Savcili small town, 911 m a.s.l., 39° 13′ 684″N, 33° 41′ 034″E, on dead stem of *Juglans regia* (Juglandaceae), 8 June 2012, Faruk Selcuk (MFLU 17-0523 holotype); ibid. (isotype in HKAS), ex-type living culture MFLUCC 18-1137.

GenBank accession numbers: LSU: MN244171, SSU: MN244177, ITS: MN244201

Notes – The new isolate *Leptosphaeria regiae* was obtained from dead stem of *Juglans regia*. In the NCBI BLASTn search of ITS sequence *L. regiae* has a closest match with *L. sclerotioides* (Preuss ex Sacc.) de Gruyter et al. (2013) (LP7-MRL) with identities 477/492 (97%) and 1% gaps. In our multigene phylogenetic analysis, *L. regiae* clusters close to *L. slovacica* (CBS 125975) with strong bootstrap support (100% ML, 1.00 PP). A comparison of 528 base pairs across the ITS (+5.8S) regions shows 71 (11.9%) base pair differences between *L. regiae* and *L. slovacica*. Morphologically, *L. regiae* differs from *L. slovacica* in its smaller ascospores (15–18 μm vs. 18–22 μm). We therefore, introduce *Leptosphaeria regiae* as a new species in the genus *Leptosphaeria* based on differences in morphology and DNA sequence data.

**FIGURE 3:**
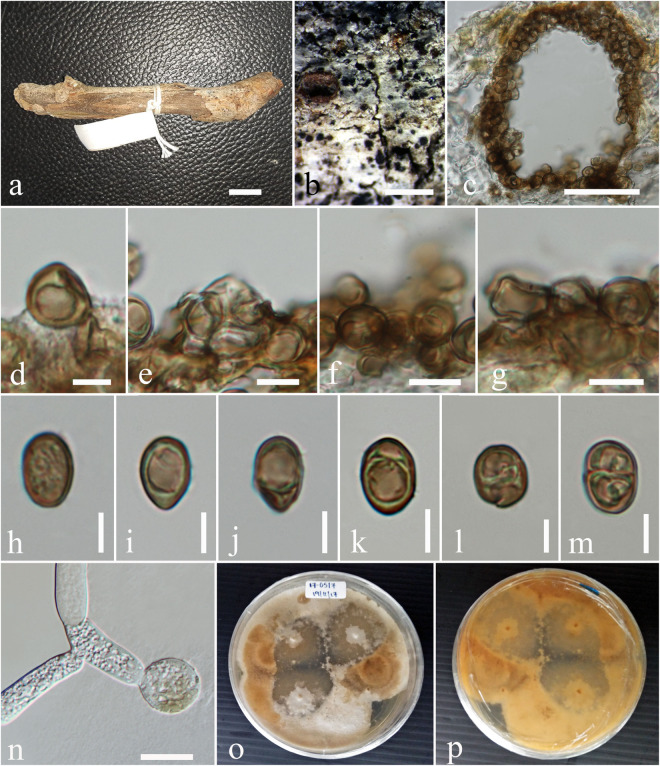
*Neomicrosphaeropsis juglandis* (MFLU 17-0517, holotype). **(a,b)** Appearance of conidiomata on host surface. **(c)** Vertical section through conidioma. **(d–g)** Conidiogenous cells and developing conidia. **(h–m)** Conidia. **(n)** Germinating conidia. **(o,p)** Culture characters on MEA (**o:** Above view; **p:** Reverse view). Scale bars: **(a,b)** = 1000 μm, **(c)** = 50 μm, **(d)** = 5 μm, **(e–g)** = 10 μm, **(h–m)** = 5 μm, **(n)** = 15 μm.

***Neomicrosphaeropsis juglandis*** D. Pem, Selcuk F, Jeewon & K.D. Hyde, **sp. nov**. Figure 3

[urn:lsid:indexfungorum.org:names: 556688]
Facesoffungi number– FoF 06211
Etymology – Name reflects the host from which the fungus was isolated
Holotype – MFLU 17-0517

*Saprobic* on stems of *Juglans regia*. **Sexual morph:** Undetermined. **Asexual morph:** *Conidiomata* 158–189 μm high × 172–228 μm diam. ($\bar{x}$ = 170.6 × 202.9 μm, *n* = 10), pycnidial, scattered, solitary, aggregated or gregarious, immersed, slightly erumpent, black, globose to subglobose, uni- to bi-loculate, non-ostiolate. *Conidiomatal wall* 10–20 μm wide comprising light to dark brown, thick-walled cells of *textura angularis*. *Conidiophores* reduced to conidiogenous cells. *Conidiogenous cells* enteroblastic, phialidic, light brown, integrated, smooth. *Conidia* 8–11 × 6–7 μm ($\bar{x}$ = 9.8 × 6.9 μm, *n* = 50), yellowish or greenish brown, aseptate, obovoid to ellipsoidal, smooth-walled, sometimes guttulate.

Culture characteristics – Colonies growing on MEA, reaching a diameter of 25 mm after 7 days at 25°C, circular to irregular, flat to slightly raised, mycelium medium sparse, surface initially white, becoming pale saffron to pale white, reverse dark-gray with whitish edge, smooth at surface with entire to slightly filamentous edge, thinly hairy.

Material examined – TURKEY, The Middle Kizilirmak river-basin, Kirikkale province, Delice district, Çerikli small town, 682 ma.s.l., 39° 53′ 689″N, 33° 59′ 769″E, on dead aerial stems of *Juglans regia* L. (Juglandaceae), 8 August 2012, Faruk Selcuk (MFLU 17–0517, holotype); ibid. (isotype in HKAS), ex-type living culture MFLUCC 18-0795, DSM 109836.

GenBank accession numbers – LSU: MN244206, SSU: MN244183, ITS: MN244223, BTUB: MN871955.

Notes – Our new taxon *Neomicrosphaeropsis juglandis* is characterized by large, aseptate conidia with a unique yellowish or greenish brown color and measures 8–11 × 6–7 μm, compared to *N. italica* (3.6–6.2 μm × 2.9–4.6 μm), the type species of the genus *Neomicrosphaeropsis*. The ITS sequence comparison of *N. juglandis* with *N. tamaricicola*, *N. italica* and *N. rossica* reveals a difference of 0.8% (5 base pairs difference). However, strong evidence to support *N. juglandis* as a new species comes from the comparison of the RPB2 gene of our new species, *N. juglandis* to that of *N. italica* which shows a pairwise difference of 1.6% as well as that of β-tubulin with *N. italica* showing a difference of 2.1%. We therefore, introduce *N. juglandis* as a new species in the genus *Neomicrosphaeropsis* (Didymellaceae) based on morphological and phylogenetic evidence derived especially from protein coding genes.

**FIGURE 4:**
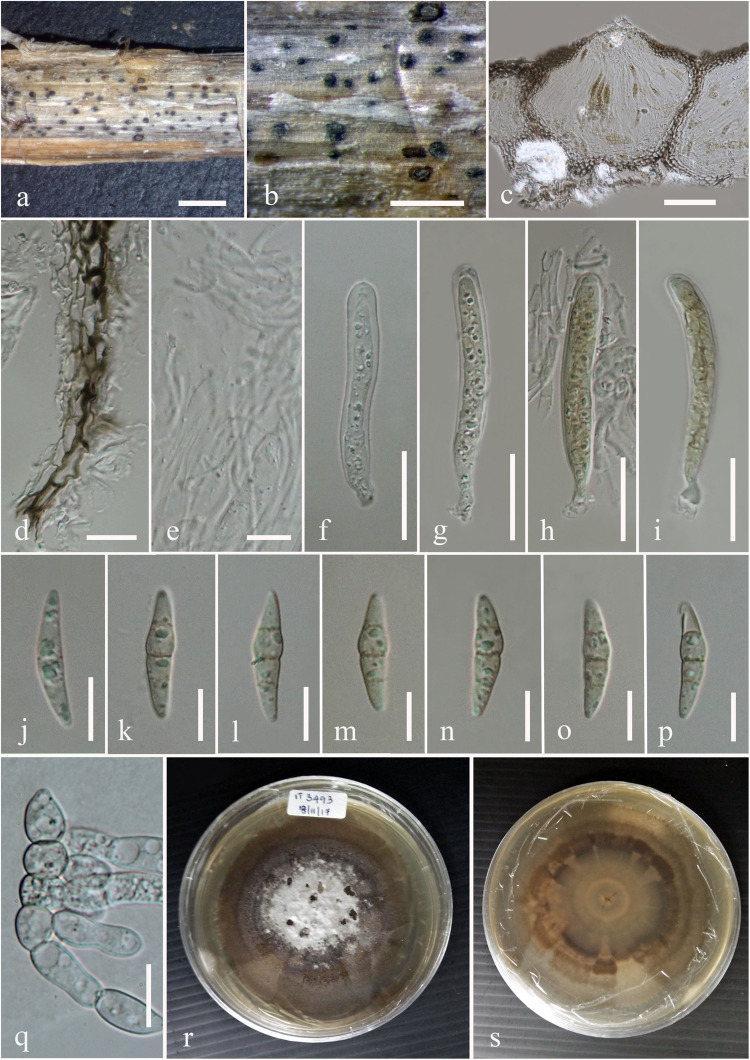
*Subplenodomus urticae* (MFLU 17-1694, holotype). **(a,b)** Appearance of ascomata on host surface. **(c)** Vertical section through the ascoma. **(d)** Peridium. **(e)** Hamathecium. **(f**–**i)** Asci. **(j–p)** Ascospores. **(q)** Germinating ascospore. **(r,s)** Culture characteristics on MEA (**r:** above view; **s:** reverse view). Scale bars: **(b–d)** = 200 μm, **(e,g,h,k)** = 50 μm, **(f)** = 5 μm, **(i,j)** = 20 μm, **(l–o)** = 10 μm.

***Subplenodomus urticae*** D. Pem, Camporesi, Jeewon & K.D. Hyde, **sp. nov.** Figure 4

[urn:lsid:indexfungorum.org:names: 557057]
Facesoffungi Number: FoF 06855
Etymology – Name reflects the host from which the fungus was isolated
Holotype – MFLU 17-1694

*Saprobic* on dead stem of *Urtica dioica*. **Sexual morph:** *Ascomata* 98–162 μm high, 111–200 μm diam., solitary or gregarious, superficial or semi-immersed on host tissue, visible as black spots on host surface, dark brown to black, papillate. *Ostiole* 29–32 × 48–66 μm, smooth, ostiolar canal filled with periphyses. *Peridium* 10–28 μm wide, comprising two cell types, outer layer composed of large, heavily pigmented, thick-walled cells of *textura angularis*, inner layer composed of hyaline cells of *textura prismatica*. *Hamathecium* comprising numerous, long, 1.7–2.4 μm ($\bar{x}$ = 2.2 μm, *n* = 50) wide, broad, transversely septate, branched, cellular pseudoparaphyses. *Asci* 44–65 × 7.3–10.7 μm ($\bar{x}$ = 58.4 × 8.9 μm, *n* = 30), 8-spored, bitunicate, numerous, cylindrical to cylindric-clavate, short pedicellate, apically rounded, with indistinct ocular chamber. *Ascospores* 19–24 × 4.4–5.6 μm ($\bar{x}$ = 21.9 × 5.2 μm, *n* = 30), overlapping biseriate, hyaline when immature, becoming yellowish brown to brown at maturity, ellipsoidal to broadly fusiform, tapering at the ends, 3–septate, constricted at septum, widest at second septum, smooth-walled. **Asexual morph:** Undetermined.

Culture characteristics – Circular, surface rough, entire edge, in the middle powdery, on the edge thinly hairy, margin well-defined and slightly radiating, white and slightly raised in the middle, greenish gray at the edge; reverse white in the middle, strongly radiating, cracking the media, greenish-gray at the edges.

Material examined – ITALY, near Balze – Verghereto [province of Forlì-Cesena (FC)], on stem of *Urtica dioica* (Urticaceae), 19 September 2017, Erio Camporesi (MFLU 17-1694, holotype), ibid. (isotype in HKAS), ex-type living culture MFLUCC 17-2311.

GenBank accession numbers – LSU: MN597995, SSU: MN597997, ITS: MN597998.

Notes – *Subplenodomus urticae* was collected from dead stem of *Urtica dioica*. Morphologically, the present collection matches the description of *S. galiicola* and *S. iridicola* in having broad cylindrical, with club-shaped pedicel. However, *S. urticae* is distinct from *S. galiicola* in having smaller ascomata (98–162 × 111–200 μm vs. 254–285 × 311–314 μm), smaller ostiole (29–32 × 48–66 μm vs. 70–98 × 98–117 μm), shorter asci (44–65 × 7.3–10.7 μm vs. 66–120 × 12–17 μm) and smaller ascospores (19–24 × 4.4–5.6 μm vs. 30–40 × 6–9 μm. Phylogenetically, *S. urticae* forms an independent lineage distinct from *S. iridicola* and other *Subplenodomus* species. *Subplenodomus urticae* differs from *S. iridicola* in having shorter asci (44–65 × 7.3–10.7 μm vs. 80–100 × 10–15 μm) and shorter ascospores (19–24 × 4.4–5.6 μm vs. 21–25 × 6–7 μm). Other species of *Subplenodomus* are in their asexual morph and thus cannot be compared. A comparison of 528 ITS (+5.8S) nucleotides between *S. urticae* and *S. galiicola* shows 57 (9.5%) base pair difference while that of *S. urticae* and *S. iridicola* shows 69 (13.1%) base pair difference. Thus, a new taxon is introduced as *S. urticae* based on the recommendations provided by Jeewon and Hyde (2016).

### Phylogenetic Analyses

**FIGURE 5:**
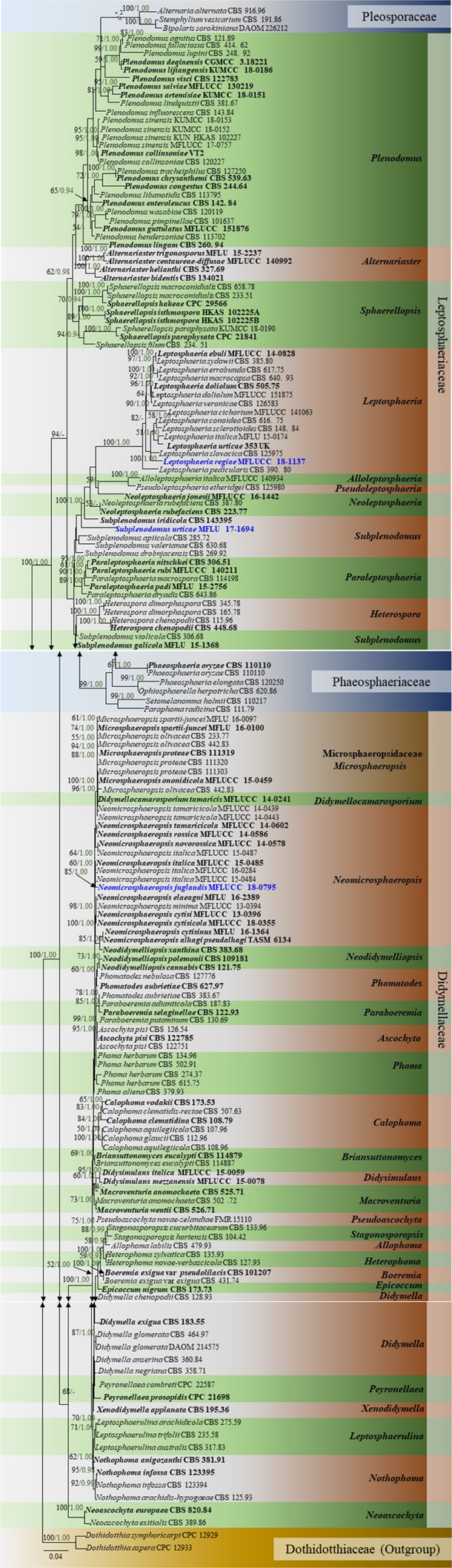
Phylogram generated from maximum likelihood and Bayesian analysis based on combined LSU, ITS, SSU, and β-tubulin sequence data retrieved from GenBank. Related sequences were referred to Chen et al. (2015), Thambugala et al. (2016), and Phookamsak et al. (2019). One hundred and sixty-nine different taxa are included in the combined genes sequence analyses which comprised 2528 characters (LSU: 1–899, ITS: 900–1510, SSU: 1511–2515, β-tubulin: 2516–2878) including gaps. *Dothidotthia symphoricarpi* (CPC 12929) and *Neodothidotthia negundinicola* (CPC 12933) are used as the out-group taxa. Maximum likelihood (ML) analysis was conducted in the CIPRES Science Gateway V.3.3. The best sorting RAxML tree with a final likelihood value of -25154.560152 is presented. Estimated base frequencies were as follows: A = 0.242800, C = 0.228484, G = 0.270168, T = 0.258548; substitution rates AC = 1.463454, AG = 3.304206, AT = 1.844561, CG = 0.804481, CT = 7.244176, GT = 1.000000; gamma distribution shape parameter α = 0.629245; proportion of invariant 0.688986. ML bootstrap values ≥50% are given as the first set of numbers and approximate likelihood-ratio test (aLRT) ≥0.90 values as the second set of numbers above the nodes. Voucher/strain numbers are given after the taxon names, the one from type material are indicated in bold face. The new taxa are given in bold and blue. The bar length indicates the number of nucleotide substitutions per site.

In the multi-locus phylogeny inferred from the combined dataset of LSU, ITS, SSU, and β-tubulin, several well-supported clades can be recognized which are used for the delimitation of the 10 genera namely, *Plenodomus*, *Alternariaster*, *Sphaerellopsis*, *Leptosphaeria*, *Alloleptosphaeria*, *Pseudoleptosphaeria*, *Subplenodomus*, *Paraleptosphaeria*, and *Heterospora* (Figure 5). The genus *Plenodomus* forms a well-supported clade within the family Leptosphaeriaceae and comprised 26 strains as well as the type species *Plenodomus lingam* (Tode: Fr.) Höhn. The genus *Alternariaster* also forms a well-supported clade sister to the *Plenodomus* clade and included four strains namely, *A. trigonosporus*, *A. centaureae-diffusae*, *A. bidentis* along with *A. helianthi*, the type species of the genus *Alternariaster*. *Sphaerellopsis* strains together with the type strain, *Sphaerellopsis filum* (Biv.) B. Sutton clustered in a distinct clade, fully supported in all analyses (89% ML, 1.00 PP). *Leptosphaeria sensu stricto* forms a well-supported clade in the family Leptosphaeriaceae comprising *L. doliolum* strains, the type species, strains of 12 other species along with the new species *Leptosphaeria regiae* (MFLUCC 18-1137). Our new taxon, *L. regiae* is close to *L. slovacica* (CBS 125975). The monotypic genera *Alloleptosphaeria* and *Pseudoleptosphaeria* form a distinct clade sister to each other and close to the genus *Neoleptosphaeria* which comprise three strains with *N. rubefaciens* as type species. Our new taxon, *Subplenodomus urticae* constitutes an independent lineage and fits within the genus *Subplenodomus*. The genus *Paraleptosphaeria* forms a well-supported monophyletic clade (89% ML, 1.00 PP) with four strains together with the type species *Paraleptosphaeria nitschkei*. The new species *Neomicrosphaeropsis juglandis* clusters in the family Didymellaceae with moderate support (85% ML, 1.00 PP). The multigene analyses show that *N. juglandis* (MFLUCC 18-0795) is phylogenetically related to species in the genus *Neomicrosphaeropsis* in particular to *N. italica* (Figure 5). We therefore, describe the three taxa as new based on the recommendations outlined by Jeewon and Hyde (2016).

## Discussion

Several recent papers have described saprobic fungi from the class Dothideomycetes from different hosts across the world (Pem et al., 2018, 2019a,b; Hyde et al., 2019, 2020; Phookamsak et al., 2018, 2019). This study reports on three species that are new to science collected from Turkey and Italy. They are *Leptosphaeria regiae*, *Subplenodomus urticae* from the family Leptopshaeriaceae and *Neomicrosphaeropsis juglandis* from the family Didymellaceae. Both Leptosphaeriaceae and Didymellaceae are highly diverse family with more than hundred species discovered during the last 10 years.

*Leptosphaeria regiae* forms a distinct lineage basal to *L. slovacica* (CBS 125975). Phylogeny recovered herein depict a close association of *Leptosphaeria regiae* to *L. slovacica* but the affinities of the latter with other species is obscure. Despite a close phylogenetic link, these two species are morphologically different. *Leptosphaeria regiae* differs from *L. slovacica* in having relatively shorter ascospores (15–18 μm vs. 18–22 μm) (de Gruyter et al., 2013). *Leptosphaeria pedicularis* is in its asexual state characterized by black globose perithecia, short ostiole and hyaline cylindrical conidia and cannot be morphologically compared to *L. regiae*. Comparison of the ITS sequences of these species shows 6.7% (*L. regiae* vs. *L. slovacica*) and 7.3% (*L. regiae* vs. *L. pedicularis*) nucleotides differences, respectively. *Leptosphaeria regiae* seems to be most closely allied to *L. cichorium* by resemblance of general morphological features such as superficial or semi-immersed globose to subglobose ascomata, peridium of schleroplectenchymatous cells and yellowish brown, fusoid, 3-septate ascospores (Ariyawansa et al., 2015). However, *L. regiae* has longer asci (99–130 × 9–10 μm vs. 71–115 × 5–8 μm) compared to *L. cichorium*. A pairwise comparison of 523 ITS (+5.8S) sequence data reveals 34 (6.8%) base pair differences between *L. regiae* and *L. cichorium* which confirms the two species as distinct. *Leptosphaeria regiae* also resembles *L. italica* in sharing superficial or semi-immersed globose ascomata and fusiform ascospores while the former differs in having longer asci (99–130 × 9–10 μm vs. 60–112 μm × 7–12 μm) (Dayarathne et al., 2015). Furthermore, *L. italica* occurs on *Rhamnus alpinus* in the Province of Forlì-Cesena in Italy while *L. regiae* was found on *Juglans regia* in the Çorum province of Turkey and they are phylogenetically apart (Figure 5). It is also worth to compare the new species *L. regiae* to that of *L. doliolum*, the type species of the genus *Leptosphaeria*. The former has shorter asci (99–130 × 9–10 μm vs. 105–150 × 7–10 μm) and smaller ascospores (15–18 × 6–7 μm vs. 25–30 × 4–6 μm) compared to *L. doliolum* which was recorded on dead stem in England (Shearer et al., 1990; Liu et al., 2015). Comparison of ITS sequence data between *L. regiae* and *L. doliolum* shows 4.4% base pair differences and these two species are far apart in the phylogenetic tree. Likewise, our new species *L. regiae* also bears morphological resemblance to *L. urticae* in having cylindric-clavate, short pedicellate asci but markedly differs from *L. urticae* in having shorter ascospores (15–18 × 6–7 μm vs. 35–40 × 4–6 μm) and in the number of ascospore septa (3 vs. 8–9) (Phookamsak et al., 2019). Moreover, the multi-locus phylogenetic study demonstrates that both species could be clearly differentiated with 25 (5.1%) ITS nucleotides differences between them. *Leptosphaeria regiae* also shares similarities to *L. ebuli* in having cylindrical to cylindric-clavate, short pedicellate asci. However, *L. regiae* has larger ascomata (315–377 × 364–410 μm vs. 226–396 × 241–251 μm) and shorter ascospores (15–18 × 6–7 vs. 23–28 × 4–5 μm) compared to *L. ebuli* (Liu et al., 2015). *Leptosphaeria regiae* and *L. ebuli* are phylogenetically distant and ITS DNA sequence comparison reveals 28 (5.9%) base pair differences. There are 605 estimated species in the genus *Leptosphaeria* but only 15 species have DNA sequence data (Species Fungorum, 2020). A synopsis of all recognized species having molecular data are provided in Table 2.

**TABLE 2:** Synopsis of *Leptosphaeria* species having DNA sequence data in GenBank.

*Leptosphaeria* species	Country	Sexual morph (μm)						Asexual morph (μm)			References
		Host	Ascomata	Peridium	Asci	Ascospores	Septate	Conidiomata	Conidiogeneous cells	Conidia	
*Leptosphaeria cichorium*	Italy	*Cichorium intybus*	206–240 × 251–363	2.5–7.5	71–115 × 5–8	11–20 × 3– 6	3-septate	189–200 × 196–220	2–5 × 2–4	3–6 × 1–3	Ariyawansa et al., 2015
*Leptosphaeria conoidea*	Italy	stems of *angelica* (Apiaceae)			90 × 5–5.5	15–20 × 4					Saccardo, 1875
*Leptosphaeria doliolum*	England	dead stem	340–460 × 360–500	85–110	105–150 × 7–10	25–30 × 4–6	3-septate	Undetermined.			Ariyawansa et al., 2015
*Leptosphaeria ebuli*	Italy	*Sambucus ebulus* (Adoxaceae)	226–396 × 241–251	24–26	80–109 × 8–9	23–28 × 4–5	3-septate	Undetermined.			Liu et al., 2015
*Leptosphaeria errabunda*	Netherlands	*Delphinium* sp. (Ranunculaceae)	Description not available								de Gruyter et al., 2013
*Leptosphaeria italica*	Italy	*Rhamnus alpinus* L. ssp. Fallax (Boiss.) Marie and Petitmangin (Rhamnaceae)	285–294 × 248–260	38–40	60–112 × 7–12	12–18 × 4–6	3-septate	Undetermined.			Dayarathne et al., 2015
*Leptosphaeria macrocapsa*	Netherlands	*Mercurialis perennis* (Euphorbiaceae)									de Gruyter et al., 2013
*Leptosphaeria pedicularis*	Switzerland	*Pedicularis sp.* (Scrophulariaceae)	Description not available								de Gruyter et al., 2013
***Leptosphaeria regiae***	Turkey	*Juglans regia* (Juglandaceae)	315–377 × 364–410	41–50	99–130 × 9–10	15–18 × 6–7	3-septate				This study
*Leptosphaeria sclerotioides*	Canada	*Medicago sativa* (Fabaceae)								5–6 × 2	de Gruyter et al., 2013
*Leptosphaeria slovacica*	Czech Republic	*Ballota nigra* (Lamiaceae)	Description not available								de Gruyter et al., 2013
*Leptosphaeria sydowii*	Switzerland, Netherlands	*Papaver rhoeas* (Papaveraceae), *Senecio Jacobaea* (Asteraceae)	Description not available								de Gruyter et al., 2013
*Leptosphaeria urticae*	England	*Urtica dioica* (Urticaceae)	100–130 × 70–110	25–50	60–140 × 9.9–11	35–40 × 4–6	(8–)9-septate				Phookamsak et al., 2019
*Leptosphaeria veronicae*	Netherlands	stem of *Veronica* “Shirley Blue” (Scrophulariaceae)	Description not available								de Gruyter et al., 2013

*New species described in this study are indicated in bold.*

There are six morphological species in the genus *Subplenodomus* (Species Fungorum, 2020) and all six species are described based on DNA sequence data (Tibpromma et al., 2017; Crous et al., 2018). The new species *Subplenodomus urticae* is morphologically similar to *S. iridicola* in sharing superficial or semi-immersed black ascomata and cylindrical asci with club-shaped pedicel but distinct in that the latter was described from *Iris* sp. (Iridaceae) from UK and has larger ascospores (21–25 × 5–7 μm vs. 19–24 × 4.4–5.6 μm) (Crous et al., 2018). A synopsis of *Subplenodomus* species is provided in Table 3. Phylogenetically, *S. urticae* clusters in the genus *Subplenodomus* basal to *S. iridicola*. *Subplenodomus violicola* is the type species of *Subplenodomus* and was established by de Gruyter et al. (2013). Since then, five additional species have been described in the genus. *Subplenodomus urticae* differs from *S. iridicola* by 13.1% nucleotide differences in the ITS regions. In our multi-gene analysis, the affinities of *Subplenodomus* corroborates those reported by previous studies (Schoch et al., 2009; Zhang et al., 2012; Crous et al., 2018; Phookamsak et al., 2019). *Subplenodomus apiicola*, *S. drobnjacensis*, *S. valerianae*, and *S. violicola* all produce pycnidia with an elongated neck. The pycnidial wall is pseudoparenchymatous. The new species *S. urticae* is unique and well-distinct among all the species reported in the genus *Subplenodomus*. *Subplenodomus urticae* is the first *Subplenodomus* species reported from *Urtica dioica* (Urticaceae) and is unique in having shorter cylindrical asci as well as ascospores compared to other species of *Subplenodomus*.

**TABLE 3:** Synopsis of *Subplenodomus* species having DNA sequence data in GenBank.

*Subplenodomus* species	Host	Country	Ascomata	Ostioles	Peridium	Asci	Ascospores	References
*Subplenodomus apiicola*	*Apium graveolens* var. *rapaceum* (Apiaceae)	Germany	Description not available					de Gruyter et al., 2013
*Subplenodomus drobnjacensis*	*Gentiana makinoi* “Royal Blue” (Gentianaceae)	Netherlands	Description not available					de Gruyter et al., 2013
*Subplenodomus galiicola*	*Galium* sp. (Rubiaceae)	Italy	254–285 × 311–314	70–98 × 98–117	32–60	66–120 × 12– 17	30–40 × 6–9	Tibpromma et al., 2017
*Subplenodomus iridicola*	*Iris* sp. (Iridaceae)	UK	150–250 diam	20–30 μm diam		80–100 × 10–15	(19–) 21–25 (–27) × (5–) 6 (–7) μm	Crous et al., 2018
*Subplenodomus valerianae*	*Valeriana* phu (Valerianaceae)	Netherlands	Description not available					de Gruyter et al., 2013
*Subplenodomus violicola*	*Viola tricolor* (Violaceae)	Netherlands	Description not available					de Gruyter et al., 2013
***Subplenodomus urticae***	*Urtica dioica* (Urticaceae)	Italy	98–162 × 111–200	29–32 × 48–66	10–28	44–65 × 7.3–10.7	19–24 × 4.4–5.6	This study

*New species described in this study are indicated in bold.*

Our new species *Neomicrosphaeropsis juglandis* is an independent lineage close to *N. italica.* Among the several genes regions analyzed, it was noted that β-tubulin DNA sequence data generated relatively well-resolved topologies to support intergeneric relationships within the Didymellaceae and particularly in connection with *Neomicrosphaeropsis* (data not shown). Our new taxon is an addition to *Neomicrosphaeropsis* and is also the first record of the genus on *Juglans regia* in Turkey. A synopsis of the asexual morph of existing species of *Neomicrosphaeropsis* is provided in Table 4. Our new taxon is unique in that it produces larger aseptate conidia compared to other *Neomicrosphaeropsis* species and has been reported from a different host. Among the phenotypically diverse species, the genus *Neomicrosphaeropsis* as well as *Didymellocamarosporium* in Didymellaceae produce pigmented, muriform spores (Hyde et al., 2016; Thambugala et al., 2016). One interesting finding is also that *Neomicrosphaeropsis cytisi, N. cytisicola and N. cytisina* are morphologically similar with conidial measurement ranging between 4–7.9 × 2.5–3.5 μm and all of them were isolated from *Cytisus* sp. (Fabaceae). The authors differentiate the two species based on size and form of conidiomata. However, it is highly probable that *N. cytisi, N. Cytisicola*, and *N. cytisina* are all same species as the ITS sequences of the three species are same (no base pair difference). RPB2 comparison of *N. cytisi* and *N. cytisicola* shows only three base pair difference across 1089 nucleotides examined. No TEF and BTUB sequences are available for the three species for comparison. Likewise, *N. tamaricicola*, *N. rossica, N. Novorossica*, and *N. italica* have been isolated from *Tamarix* species and are morphologically similar with conidial measurements ranging from 3.5–6.6 × 2.5–4.6 μm. Comparison of RPB2 and BTUB gene sequences between *N. italica* and *N. tamaricola* shows no base pair difference. However, comparison of TEF gene sequences between *N. italica* to *N. rossica* and *N. tamaricicola* shows 7 or 1.0% and 9 or 1.3% base pair differences, respectively, while there was no base pair differences between *N. rossica* and *N. tamaricicola*. It can be possible that some of these species are the same and need to be synonymised in future studies. In our phylogenetic analyses, *N. minima* is closely related to *N. cytisi* with strong bootstrap support. Comparison of available ITS and TEF sequences of *N. minima* and *N. cytisi* reveals zero base pair difference, however, there are some slight differences in conidial sizes (*N. minima*: 2.8–5.4 × 9.2–3.6 μm vs. *N. cytisi:* 4.5–7.9 × 3–5 μm). *Neomicrosphaeropsis minima* also differs from *N. cytisi* in conidiomatal size (*N. minima*: 60–80 μm diam., 60–95 μm high vs. *N. cytisi:* 75–155 μm diam., 75–130 μm high) and were isolated from different hosts. Whether these species are distinct and merit a specific taxonomic rank warrant further investigations given that minor conidial and conidiomatal size differences could vary under cultural and host conditions. Recollecting and sequencing of more *coniothyrium*-like and more fungi similar to *Neomicrosphaeropsis* from different geographical regions are also essential to clarify the placement of species and to infer relationships in *Neomicrosphaeropsis* and *Neomicrosphaeropsis*-like genera within the Didymellaceae (Pleosporales).

**TABLE 4:** Synopsis of the asexual morph of existing species of *Neomicrosphaeropsis*.

*Neomicrosphaeropsis* species	Asexual morph		Hosts	Country	References
	Conidiogenous cells (μ m)	Conidial size (μ m)			
*Neomicrosphaeropsis alhagi-pseudalhagi*	7–12 × 8–10	30–45 × 18–22	*Alhagi pseudalhagi* (M. Bieb.)Desv (Fabaceae)	Uzbekistan	Wanasinghe et al., 2018
*Neomicrosphaeropsis cytisi*	2– 4 × 9 3.5–6	4.5–7.9 × 3–5	*Cytisus* sp. (Fabaceae)	Italy	Hyde et al., 2016
*Neomicrosphaeropsis cytisicola*	–	4–7 × 2.5–3.5	*Cytisus* sp. (Fabaceae)	Italy	Wanasinghe et al., 2018
*Neomicrosphaeropsis cytisina*	1–2 × 1–2	5–7.9 × 3–5	*Cytisus scoparius L.* (Fabaceae)	Italy	Wanasinghe et al., 2018
*Neomicrosphaeropsis elaeagni*	–	16-20 × 7-9	*Elaeagnus angustifolia* L. (Elaeagnaceae)	Russia	Wanasinghe et al., 2018
*Neomicrosphaeropsis italica*	2.5–5 × 1.8–3.1	3.6–6.2 × 2.9–4.6	*Tamarix* sp. (Tamaricaceae)	Italy	Thambugala et al., 2016
*Neomicrosphaeropsis minima*	2.6–5.5 × 9.2–3.5	2.8–5.4 × 9 2–3.6	*Verbascum* sp. (Scrophulariaceae)	Italy	Hyde et al., 2016
*Neomicrosphaeropsis novorossica*	2.6–4.2 × 2–3.4	4.3–7.5 × 3.6–5.1	*Tamarix ramosissima* Ledeb. (Tamaricaceae)	Russia	Thambugala et al., 2016
***Neomicrosphaeropsis juglandis***	–	8–11 × 6–7	*Juglans regiae* (Juglandaceae)	Turkey	This study.
*Neomicrosphaeropsis rossica*	3–4.6 × 2–4	4.4–5.7 × 2.9–3.9	*Tamarix ramosissima* Ledeb. (Tamaricaceae)	Russia	Thambugala et al., 2016
*Neomicrosphaeropsis tamaricicola*	2–4 × 1.6–3.2	3.5–6.6 × 2.5–3.4	*Tamarix gallica* L. (Tamaricaceae)	Italy	Thambugala et al., 2016

*New species described in this study are indicated in bold.*

## Data Availability Statement

The datasets generated for this study can be found in the sequences retrieved from the 28S rRNA, Internal transcribed spacer rDNA region (ITS1, 5.8S rDNA, and ITS2), 18S rRNA and beta-tubulin (TUB) gene sequencing and were deposited at GenBank-NCBI under the nucleotide accession number as follows: MN244171, MN244201, MN244177 (*Leptosphaeria regiae*), MN244206, MN244223, MN244183, MN871954 (*Neomicrosphaeropsis juglandis*), MN597995, MN597998, MN597997 (*Subplenodomus urticae*).

## Author Contributions

DP, RJ, and KH designed the study. FS did the sample collections. DP and RJ were involved in the phylogenetic analyses. SL contributed to the research funds. All authors contributed to the article and approved the submitted version.

## Conflict of Interest

The authors declare that the research was conducted in the absence of any commercial or financial relationships that could be construed as a potential conflict of interest.

## Footnotes

1 http://www.indexfungorum.org

2 http://www.treebase.org/

3 http://www.ncbi.nlm.nih.gov

4 http://mafft.cbrc.jp/alignment/server/index.html

5 http://tree.bio.ed.ac.uk/software/figtree/
